# Significance of Normalization on Anatomical MRI Measures in Predicting Alzheimer's Disease

**DOI:** 10.1155/2014/541802

**Published:** 2014-01-06

**Authors:** Qi Zhou, Mohammed Goryawala, Mercedes Cabrerizo, Warren Barker, Ranjan Duara, Malek Adjouadi

**Affiliations:** ^1^Department of Electrical Engineering at the Florida International University, Miami, FL 33174, USA; ^2^Wien Center for Alzheimer's Disease and Memory Disorders, Mount Sinai Medical Center, Miami Beach, FL 33140, USA; ^3^Florida International University, 10555 West Flagler Street, EC 2672, Miami, FL 33174, USA

## Abstract

This study establishes a new approach for combining neuroimaging and neuropsychological measures for an optimal decisional space to classify subjects with Alzheimer's disease (AD). This approach relies on a multivariate feature selection method with different MRI normalization techniques. Subcortical volume, cortical thickness, and surface area measures are obtained using MRIs from 189 participants (129 normal controls and 60 AD patients). Statistically significant variables were selected for each combination model to construct a multidimensional space for classification. Different normalization approaches were explored to gauge the effect on classification performance using a support vector machine classifier. Results indicate that the Mini-mental state examination (MMSE) measure is most discriminative among single-measure models, while subcortical volume combined with MMSE is the most effective multivariate model for AD classification. The study demonstrates that subcortical volumes need not be normalized, whereas cortical thickness should be normalized either by intracranial volume or mean thickness, and surface area is a weak indicator of AD with and without normalization. On the significant brain regions, a nearly perfect symmetry is observed for subcortical volumes and cortical thickness, and a significant reduction in thickness is particularly seen in the temporal lobe, which is associated with brain deficits characterizing AD.

## 1. Introduction

Alzheimer's disease (AD) is a neurodegenerative disease and is the most common form of dementia. Estimates from the Alzheimer Association as of March 2012 indicate that 5.4 million Americans are diagnosed with AD, and over 95% of this population are 65 years of age or older. Also, nearly half of the population over 85 years of age is affected by AD [1]. The worldwide societal cost of dementia is enormous, which is estimated to be 315.4 billion USD on the basis of a 29.3 million population diagnosed with dementia [2]. AD patients display disease-related regional cerebral atrophy, which can be distinguished from normal aging [3, 4]. In AD, atrophy is often observed in regions which are closely related to neurodegeneration. Various studies have shown that atrophy in regions like the hippocampus [5–8], amygdala [5, 9] and ventricles [10, 11] is correlated with AD. Moreover, determination of the key atrophied regions across the entire brain could be used as parameters for the delineation of AD patients from cognitively normal (CN) subjects.

Freesurfer is a popular highly automated MRI image processing software widely used to generate regional measures from MRI scans. The advantages of Freesurfer over traditional manual segmentations and measures are its high automation and independence of operator subjectivity. Freesurfer is also accurate, precise and has been tested on large cohorts of studies in AD classification research [12–15].

Mini-mental state examination (MMSE) is a neuropsychological test that is most often administered to screen patients for cognitive impairment and dementia [16]. MMSE is used to judge the severity of cognitive impairment by administrating 30 questions aimed at testing the subject's orientation to time and place, attention, and calculation capabilities, as well as response to recall, language, and complex commands. The frequent use of MMSE in clinical environments makes it interesting to investigate its discriminative power in classifying AD subjects as compared to MRI-based measures.

Important tasks to be considered in AD classification studies include the choice of parameters, the way these parameters ought to be combined, and determining the preprocessing techniques to be employed in order to enhance the prospects of classification. Two essential questions that need to be addressed for AD classification studies are (1) which regional MRI measures produced by Freesurfer are statistically significant for classification of AD subjects? and (2) which normalization approach should be employed to minimize bias due to differences in head size and brain structure in order to enhance the classification performance?

Westman and his colleagues have investigated some aspects of the aforementioned issues using a supervised multivariate data analysis using the orthogonal projections to latent structures (OPLS) model [15]. OPLS is similar to principal component analysis (PCA) as they both are linear decomposition techniques and project the original data to the found latent variables. The approach of this study is an extension of a previous study [14], which proposes constructing for each classification model an optimal decisional space using the most statistically significant variables. The number of dimensions in the classifier is determined by an incremental error analysis, which in turn defines and ranks variables on their statistical significance to be used as input to an SVM-based classification process.

In this study, single-measure models and hierarchical models with and without normalization are both examined to find the optimal model. Single measure models include one of the regional MRI measures (subcortical volume, cortical thickness and surface area) or the neuropsychological test, mini-mental state examination (MMSE). A hierarchical model combines two or more of the single-measure models to examine if the interaction augments the classification process. The specific aims of this study are, thus, to determine (1) the impact of neuropsychological test (MMSE) towards the classification; (2) the combination of regional measures and MMSE that yields the best classification performance; and (3) which normalization scheme should be employed to achieve a better classification performance.

## 2. Materials and Methods

### 2.1. Subjects

A total of 189 subjects are included in this study as shown in Table 1. All participants are from the Wien Center for Alzheimer's Disease and Memory Disorders with the Mount Sinai Medical Center, Miami Beach, FL, USA. All subjects have taken the Folstein mini-mental state examination [16] with a minimum score of 15 out of 30. The study was approved by the Mount Sinai Medical Center Institutional Review Board with informed consent provided by the subjects or legal representatives.

All subjects had (1) a neurological and medical evaluations by a physician and (2) a full battery of neuropsychological tests [17], according to the National Alzheimer's Coordinating Center protocol, and the following additional tests: the three-trial fold object memory evaluation [18] and the Hopkins Verbal Learning Test; as well as (3) a structural volumetrically acquired MRI scan of the brain. The clinical dementia rating scale (CDR-sb) was used as the index of functional ability, and the MMSE was used as the index of cognitive ability. The cognitive diagnosis was made using a combination of the physician's diagnosis and neuropsychological diagnosis. The etiological diagnosis was made by the examining physician. The diagnosis of cognitively normal controls (CN) required that the physician's diagnosis was CN and no cognitive test scores were ≥1.5 SD below age and education-corrected means. A probable AD diagnosis required a dementia syndrome and the National Institute of Neurological Disorders and Stroke (NINDS)/Alzheimer's Association criteria for AD [19].

### 2.2. MRI Scans

MRI scans were acquired on a 1.5-T machine (Siemen's Symphony, Iselin, NJ, USA, or General Electric, HDX, Milwaukee, WI, USA) using a proprietary 3D-magnetization-prepared rapid-acquisition gradient echo (3D MPRAGE) or 3D spoiled gradient echo sequences (FSPGR). Specifications for 3D MPRAGE include coronal sections with a 1.5 mm gap in thickness; section interval, 0.75 mm; TR, 2190 ms; TE, 4.38 ms; TI, 1100 ms; FA, 15°; NEX, 1; matrix, 256 × 256; FOV, 260 mm; bandwidth, 130 Hz/pixel; acquisition time, 9 minutes; and phase-encoding direction, right to left. Specifications for 3D FSPGR were the following: 140 contiguous coronal sections of 1.2 mm thickness; contiguous images with no section interval; TR, 7.8 ms; TE, 3.0 ms; inversion recovery preparation time, 450 ms; flip angle, 12°; NEX, 1; matrix, 256 × 256; FOV, 240 mm; bandwidth, 31.25 Hz/pixel; acquisition time, 6–7 minutes; and phase-encoding direction, right to left.

### 2.3. Regional Volume Segmentation and Cortical Thickness Segmentation

Freesurfer pipeline version 5.1.0 (http://surfer.nmr.mgh.harvard.edu/), widely used in AD research [12–15, 20], was applied to all the MRI scans to produce 55 volumetric variables, including 45 volumetric measures of subcortical parcellation and 10 morphometric statistics. For cortical thickness, 34 regional variables were determined for each hemisphere, resulting in 68 variables for cortical thickness measures. Also, surface area was estimated from 35 regions of the brain for each hemisphere resulting in 70 measures for the entire brain.

### 2.4. Feature Extraction and Incremental Error Analysis

All the variables in a given model are first ranked based on statistical significance between AD and CN. Following this ranking, an incremental error analysis is used whereby the SVM classifier is trained and tested adding a single variable at a time to the classifier to determine the combination of top-ranked variables that yield the optimal classification outcome. This rigorous blind feature selection technique differs from others as it does not rely on prior assumptions of regions of interest (ROI) and thus assigns equal weights to all the variables. The above process was performed on all models to compare their discriminative power and consequently identify the optimal model for AD classification. It should be noted that although regional atrophy among AD patients is what is generally sought, the statistical test considers both cases of atrophy and enlargement of these specific brain regions, since volumetric enlargement can be experienced in regions like the ventricles, which has been shown to be important in differentiating AD and its prodromal stages [7, 11, 21].

### 2.5. Normalization and Classification Experiment

To explore the effect of normalization on the classification performance, MRI measures are normalized by the widely accepted morphometric measures like intracranial volume (ICV) for regional subcortical volumes, ICV and mean cortical thickness of the subject for regional cortical thickness, and ICV and the total surface area of the subject for regional surface area. A summary of the normalization measures is presented in Table 2. ICV is derived from the MRI and is one among the 10 morphometric statistics obtained by the Freesurfer pipeline. Mean cortical thickness is estimated by averaging the thickness of all the 68 regions of the brain for each subject. Similarly, total surface area is the sum of all regional surface area measures for a given subject.

Classification was performed using a support vector machine (SVM) classifier, which is shown to be effective as a classification tool for AD [22–24]. The kernel function of the SVM used for this particular study is the Gaussian radial basis function kernel (*rbf*) with a scaling factor (*σ*) of 3. All the classification results reported here are based on a 5-fold cross validation process. Each classification experiment was run 50 times, the results of which are averaged to evaluate the performance in terms of accuracy, sensitivity, specificity, and precision.

## 3. Results and Discussions

### 3.1. Classification Performance and Model Selection

Single measure models using only one type of the regional measures or MMSE were created for subcortical volume, cortical thickness, surface area, and neuropsychological data (MMSE) for both raw and normalized data. Hierarchical models were also created by combining two or more of the single models for both raw and normalized data. Feature selection based on statistical testing was performed for all the models created. The results of models with raw data are shown in Table 3 and the results for models with normalized data are shown in Table 4. All the results display an average of 50 runs with minimum and maximum values shown in parentheses.

Results of the different models are highly consistent as results of the 50 independent repetitions of classification fall within a small range as shown by the minimum and maximum values in Tables 3 and 4. This small range is a clear indication of the replicability of results, both essential attributes in any classification process. These results also indicate that MMSE is an important factor that should be included in the classification process. Inclusion of MMSE with other measures improves significantly the classification results. For example, in the case of the optimal model, hierarchical model using subcortical volumes (SV) with the inclusion of MMSE resulted in an improvement of 9.2% as compared to using SV alone. In retrospect, an average improvement of 13.3% is seen on comparing analogous models with and without MMSE when using raw data and 12.8% when using normalized data.

The classification results given in Tables 3 and 4 show that cortical thickness should be normalized by either the mean thickness of all the measured regions or ICV, while normalizing subcortical volumes to ICV does not have any significant effect. In a recent study, Westman et al. explored the normalization effect of regional MRI measures using orthogonal partial least square to latent structures (OPLS) models and concluded that both cortical thickness and subcortical volumes should not be normalized [15]. Both studies, thus, suggest that subcortical volumes should not be normalized to ICV. The divergence is seen in the normalization of cortical thickness. This could be potentially explained by the difference of the technique being used. Westman and his colleagues used an all variables inclusive model (OPLS) and the proposed method is feature selection based. The cause might be that normalization of cortical thickness brings down the variation of all the regions in general which OPLS model rely on but enhances variation in some specific regions that feature selection method might have selected. Thus, normalization of cortical thickness depends on the processing technique used. Also, the divergence can be due to the subtle differences in the data that is used for the study.

Since some models have very close performance in terms of the 4 recorded performance metrics (accuracy, sensitivity, specificity and precision), models that give more than 90% accuracy are considered as good models and are italicized in Tables 3 and 4. Inclusion of additional measures does not guarantee a significant performance enhancement. A tradeoff exists between models with some displaying better accuracy at the cost of sensitivity and vice versa. In terms of accuracy, the model of “MMSE + SV” is the best, whereas, in terms of sensitivity, the model of “MMSE + CT (Mean)” is more appropriate.

A comparison of classification performance with recent studies in the literature is provided in Table 5. The results indicate that the proposed technique using MMSE and MRI can yield competitive classification performance as those using two or more imaging modalities or biomarkers. As Westman et al. described the concept of cost-benefits to assess the increased cost of combining biomarkers as the potential limitation [25], the proposed approach has the advantage of low cost yet high accuracy. In addition, the results in this study are based on a larger cohort than most other studies in the table.

### 3.2. Univariate Analysis of Anatomical Measures

This section investigates how normalization affects the statistical significance of the variables that are used in the classification model. The effect of normalization can be determined by observing the change in the significance of the MRI measures when normalization is carried out. To illustrate the effect of normalization approaches on the statistical significance of region of interests (ROIs), univariate analysis was performed for subcortical volumes as shown in Table 6, on surface area for left and right hemisphere, respectively, as shown in Table 7, and on cortical thickness for left and right hemispheres, respectively, as shown in Table 8. Univariate analysis was created for the two hemispheres separately for both cortical thickness and surface area in order to inspect the possible pattern differences between left and right hemisphere. In Tables 6–8, the regions of the brain for which the significance of the variable differs between raw and normalized data are bolded. Please note that only those regions which show such a behavior for both the normalization techniques are highlighted in Tables 7 and 8.

Table 6 shows that ICV normalization to the subcortical volumes does not change the statistical significance of the variables, particularly for the top-ranked variables, suggesting that normalizing subcortical volumes with ICV might not be necessary, which is consistent with the conclusion made previously that subcortical volumes are not recommended to be normalized to ICV as seen from the results provided earlier in Tables 3 and 4.

More importantly, subcortical volumes and cortical thickness show symmetry between the left and right hemispheres for the top-ranked variables as shown in Tables 6 and 8. In other words, regions of the brain that are significant towards classification of AD subject are symmetrically located on either lobes of the brain. A typical example is seen in the top 5 ranked regions according to subcortical volumes which include both the right and left hippocampus and the right and left inferior lateral ventricles.

However, Table 7 shows that for the surface area there is almost no symmetry at all between the left and right hemispheres for both the raw and normalized data. This could possibly be explained by the fact that all variables found to be significant using surface area possess a *P*-value close to the significance level threshold (0.05). Another point to be noted is that for both raw and normalized data, surface area has a smaller number of significant variables and relatively high *P*-values, indicating that surface area may be generally regarded as a weaker biomarker of AD atrophy than the other two measures which are SV and CT.

**Figure 1:**
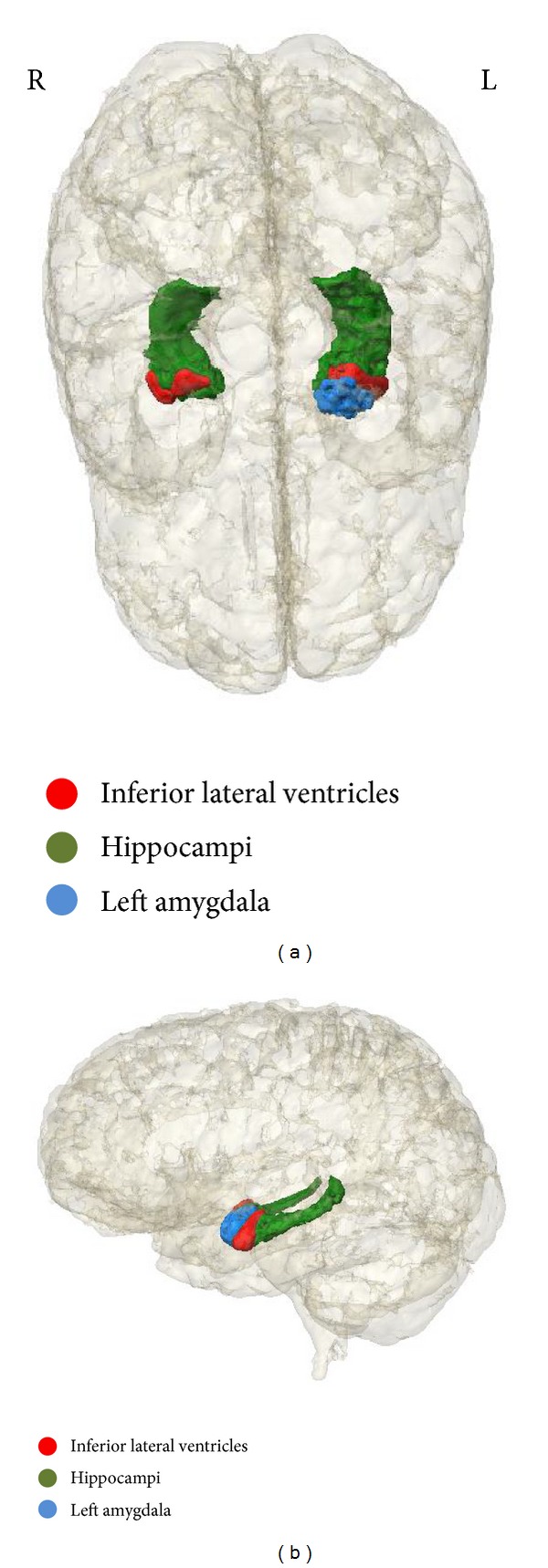
Representation of the top 5 significant subcortical volumes based on raw data in Table 6. (a) Superior view (b) Lateral view.

**Figure 2:**
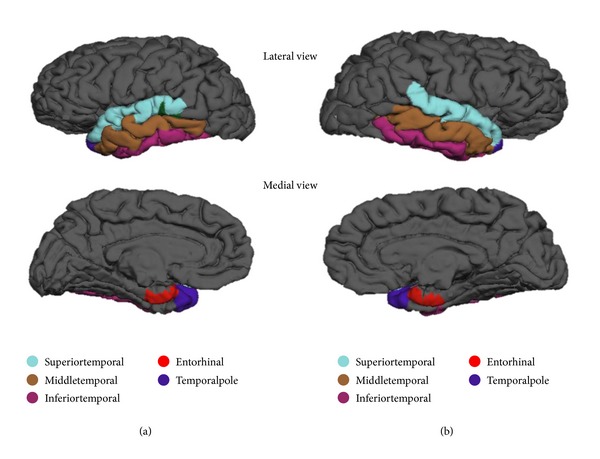
Representation of the top 5 significant cortical thickness based on raw data in Table 8. (a) Left hemisphere (b) Right hemisphere.

**Figure 3:**
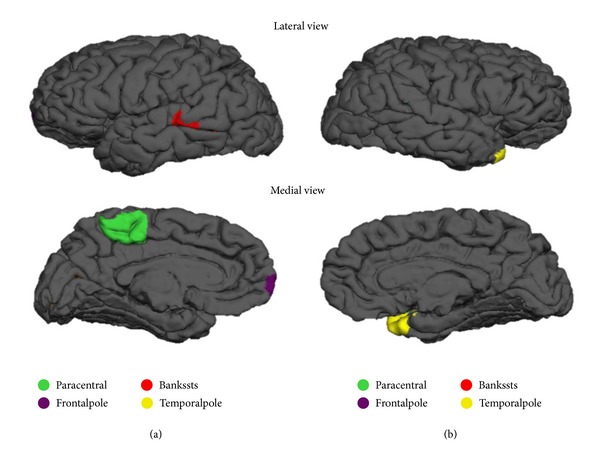
Representation of all significant surface area based on raw data in Table 7. (a) Left hemisphere (b) Right hemisphere.

**Figure 4:**
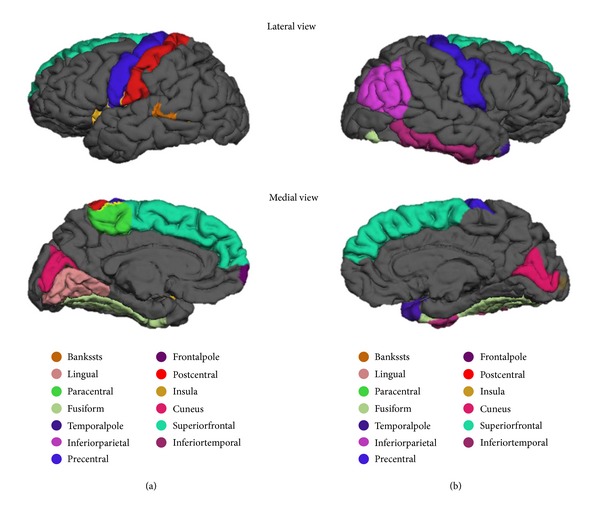
Representation of all significant surface area based on total-area normalized data on Table 7. (a) Left hemisphere (b) Right hemisphere.

The regions of the brain which are determined to be statistically significant are displayed in Figures 1–4.  Figure 1 represents the top 5 significant subcortical volumes based on raw data. Figures 2 and 3 represent the cortical regions of the brain which are found to be significant for AD classification using cortical thickness (CT) and surface area (SA) respectively on raw data.  Figure 4 illustrates the change that is seen in the significant regions of the brain when surface area normalized to the total surface area is used as a measure, as compared to raw data as shown in Figure 3.

One interesting finding about cortical thickness in Figure 2 is that most of the significant regions belong to the temporal lobe, suggesting that the temporal lobe undergoes the most significant thickness change. This is consistent with the result found by some other studies [26, 27], particularly the finding that large degree of thinning of temporal cortical thickness seen in AD while thinning is relatively reserved in normal aging [27]. The nonsymmetric atrophy pattern of surface area can be easily observed anatomically in Figures 3 and 4.

### 3.3. Spatial Distribution of Subjects under the “Best Model”

**Figure 5:**
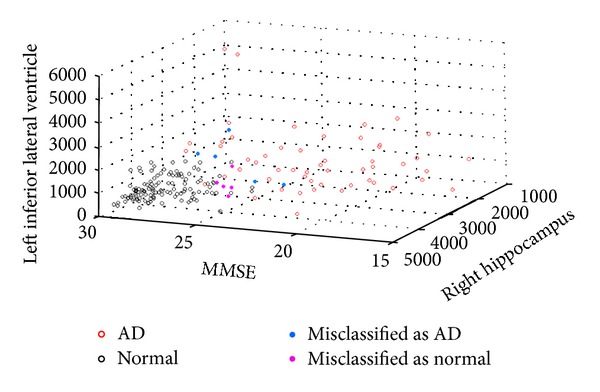
Representation of the whole dataset for the “MMSE + subcortical volume” model, for a typical classification run under this model.

Model of “MMSE + SV” without normalization gives the highest classification accuracy which utilizes the top 3 variables found within the model (i.e., MMSE, right-hippocampus volume, and left-inferior-lateral-ventricle volume). One typical distribution of the data points for this classification model is plotted in Figure 5 to show the clustering characteristics of the data when MMSE and subcortical volumes are employed. Using this optimal decisional space, it can be observed that all the normal subjects are grouped into a very compact cluster, whereas AD subjects are more sparsely distributed in context of these dimensional parameters. This indicates the complex pattern of atrophy undergoing among the AD patients, which renders the classification task extremely difficult.

### 3.4. Model Efficiency Estimation and Normalization

Variation in measures can come from many sources, including variation due to AD atrophy (*σ*_ADa_^2^), which is of primary interest for classification purposes, as well as other variation noise (*σ*_*n*_^2^) like individual difference in brain size, structure of brain regions, MRI measure error, region segmentation error, atrophy due to normal aging, and resistance to brain atrophy (e.g. cognitive reserve). Generally, the total variance can be described as follows:

(1) $$\begin{array}{c} {\sigma^{2}}_{\text{total}}=\sigma_{\text{ADa}}^{2}+\sigma_{n}^{2}, \end{array}$$

where *σ*_total_^2^ is the total variance of dataset, *σ*_ADa_^2^ stands for variance due to AD atrophy and *σ*_*n*_^2^ is the variance due to what is termed here as an overall source of noise. Also, discriminative power of a model depends on the amount of variance due to AD atrophy captured by the model used in contrast to the variance due to noise. A relevant term called discriminative power (Dp) can be estimated using

(2) $$\begin{array}{c} \text{Dp}=\frac{\tilde{\sigma_{\text{ADa}}^{2}}}{\tilde{\sigma_{n}^{2}}}, \end{array}$$

where $\tilde{\sigma_{\text{ADa}}^{2}}$ is an estimate of the variance due to AD atrophy captured by the model and $\tilde{\sigma_{n}^{2}}$ stands for the estimated variance due to noise captured by the model.

Our results, thus, show that normalization in general does not enhance the classification performance significantly, which could be explained through (2) which shows that normalization does bring down correlated noise ($\tilde{\sigma_{n}^{2}}$) experienced through brain size difference, but it also lowers the correlated variance due to AD atrophy $\tilde{(\sigma_{\text{ADa}}^{2}}$). A supporting finding of this assumption is that proportional volumes of the superior temporal cortex, expressed as a proportion of total cerebral volume, were significantly different between females and males [28], which exemplifies the fact that normalization may be intrinsically biased. A similar finding by Barnes et al. is that normalization of all volumes by head size is not adequate due to their nonproportional relationship [29]. Also, Ross et al. found that males generally have a larger overall brain size than females, and males have larger cerebral cortical volumes than females except for left parietal [30]; thus, normalization will at least bring in noise to the regions in left parietal as the regions in that area for males have a smaller size but, normalized to a larger head size. However, the Dp value could still serve as a measure of a model's performance if relevant sources of the variance are known and are quantifiable, which is not the case in most practical scenarios.

## 4. Conclusion

This paper studied the effect of normalization on the proposed statistical feature selection approach using ROIs segmented by Freesurfer and a neuropsychological test in terms of classification performance. The results shows that subcortical volume should not be normalized and surface area does not bear much discriminative information as compared to subcortical volumes or cortical thickens. Also, subcortical volumes and cortical thickness based brain maps of significant regions show symmetry between the two hemispheres which is not seen in the brain maps generated using surface area. Moreover, the feature selection method implemented on cortical thickness measures show that normalization to either ICV or mean thickness exhibits an enhancement on the classification performance, and the most pronounced changes in the cortical thickness related to AD are seen in the temporal lobe of the brain, which is shown to be related to symptoms in AD patients regarding organization, language, understanding, and so forth. A comparison of results using the optimal model which combines MMSE with subcortical volumes shows that the proposed study achieved competitive accuracy of 92.3% using fewer biomarkers, which makes it costeffective and convenient.

## Figures and Tables

**Table 1:** Demographic and neuropsychological characteristics of subjects.

	Age	Female/male	MMSE
CN (*n* = 129)	72.9 ± 6.4	92/37	28.7 ± 1.4
AD (*n* = 60)	79.5 ± 6.9	34/26	22.6 ± 3.4
*P*	<0.001	ns	<0.001

Data presented as mean ± SD where applicable.

Two-way student's *t*-test was used to test for age and MMSE and Fisher's exact test was used to test for gender.

CN: cognitively normal; AD: Alzheimer's disease; MMSE: mini-mental state examination; SD: standard deviation.

**Table 2:** Normalization measures.

MRI measure	Morphometric normalization measure
Subcortical volumes (SV)	Intracranial volume (ICV)
Cortical thickness (CT)	Intracranial volume (ICV) Mean cortical thickness
Surface area (SA)	Intracranial volume (ICV) Total surface area

**Table 3:** Classification performances on raw data.

Model	Accuracy		Sensitivity		Specificity		Precision	
MMSE	88.3	(87.3–89.4)	81.0	(76.7–81.7)	91.6	(91.5–94.6)	82.6	(81.3–87.6)
Subcortical volume (SV)	83.1	(81.5–85.2)	77.9	(75.0–80.0)	85.6	(83.0–88.4)	72.6	(69.2–77.6)
Cortical thickness (CT)	77.7	(76.2–78.9)	74.8	(73.3–76.7)	79.0	(77.4–80.7)	63.0	(59.9–68.0)
Surface area (SA)	71.4	(68.3–73.6)	58.7	(53.3–65.0)	77.2	(73.6–79.8)	55.0	(51.9–58.9)
**Average**	**80.1**		**73.1**		**83.4**		**68.3**	

Hierarchical model								

*MMSE + SV *	*92.3 *	*(90.5–93.1) *	*88.2 *	*(85.0–90.7) *	*94.2 *	*(92.3–95.3) *	*88.3 *	*(85.1–90.5) *
*MMSE + CT *	*91.4 *	*(90.4–92.6) *	*85.3 *	*(83.3–88.3) *	*94.2 *	*(93.0–95.4) *	*87.8 *	*(85.1–90.3) *
MMSE + SA	88.6	(86.3–89.5)	76.3	(71.7–78.3)	94.3	(91.5–95.4)	87.1	(81.4–89.9)
CT + SV*	83.1	(81.5–85.2)	77.9	(75.0–80.0)	85.6	(83.0–88.4)	72.6	(69.2–77.6)
SA + CT + SV*	83.1	(81.5–85.2)	77.9	(75.0–80.0)	85.6	(83.0–88.4)	72.6	(69.2–77.6)
*MMSE + SA + CT + SV∗∗ *	*92.3 *	*(90.5–93.1) *	*88.2 *	*(85.0–90.7) *	*94.2 *	*(92.3–95.3) *	*88.3 *	*(85.1–90.5) *
**Average**	**88.5**		**82.3**		**91.4**		**82.8**	

*The results of these models are the same as those of model of “SV” since the variables extracted for the decisional space are the same as those for “SV” model.

**This model gives identical results as those of the model of “MMSE + SV” since variables extracted for the decisional space are the same as those for “MMSE + SV.”

**Table 4:** Classification performances on normalized data.

Model	Accuracy		Sensitivity		Specificity		Precision	
Subcortical volume (SV)	83.5	(82.0–84.7)	74.4	(71.7–76.7)	87.7	(95.3–90.0)	75.2	(72.0–79.0)
Cortical thickness (CT)	79.0	(77.8–80.4)	78.8	(75.0–81.7)	79.2	(77.5–80.6)	64.5	(61.8–87.4)
CT (Mean)*	78.9	(77.2–80.5)	78.4	(75.0–81.7)	79.2	(75.7–80.7)	64.6	(60.9–68.4)
Surface area (SA)	72.3	(68.8–75.2)	42.6	(35.0–48.3)	86.1	(82.1–89.2)	60.4	(50.8–65.3)
SA (Area)**	72.6	(70.3–75.1)	61.2	(58.3–63.3)	77.9	(75.1–81.4)	57.4	(52.9–61.8)
**Average**	**77.3**	**67.1**	**82.02**	**64.4**	

Hierarchical model								

*MMSE + SV *	*91.7 *	*(90.0–93.1) *	*85.8 *	*(81.7–88.3) *	*94.5 *	*(93.1–95.4) *	*88.2 *	*(85.0–90.7) *
*MMSE + CT *	*91.5 *	*(89.4–93.2) *	*86.9 *	*(81.7–90.0) *	*93.6 *	*(90.8–96.1) *	*87.2 *	*(82.7–90.9) *
MMSE + CT (Mean)*	*90.3 *	*(89.2–91.1) *	*90.8 *	*(89.6–91.7) *	*90.1 *	*(88.2–91.7) *	*81.4 *	*(78.5–83.9) *
MMSE + SA	88.3	(87.3–88.9)	80.9	(76.7–81.7)	91.7	(91.4–93.8)	82.7	(81.1–86.1)
MMSE + SA (Area)**	88.6	(86.8–89.9)	80.9	(75.0–78.3)	94.2	(92.2–95.4)	86.9	(84.6–89.5)
CT + SV	83.1	(80.9–84.2)	75.8	(73.3–76.7)	86.5	(84.4–88.4)	73.3	(70.2–76.8)
CT + SA + SV	83.4	(81.0–85.7)	78.0	(75.0–80.0)	85.9	(83.0–89.1)	73.2	(68.3–69.4)
*MMSE CT + SA + SV *	*91.7 *	*(90.4–92.6) *	*86.0 *	*(83.3–90.0) *	*94.4 *	*(93.7–95.4) *	*88.4 *	*(86.5–90.2) *
**Average**	**88.6**		**83.1**		**91.3**		**82.7**	

*Scaled by the mean thickness of the all the thickness measures.

**Scaled by the total area of the all the measures.

**Table 5:** Performance comparison of different methods.

Authors	Imaging modality/biomarkers	Source of data (AD/CN)	Repetition (cross validation)	Accuracy (%)	Sensitivity (%)	Specificity (%)
Zhang et al., 2011 [20]	MRI	ADNI (51/52)	10 (10 folds)	86.2	86	86.3
Zhang et al., 2011 [20]	CSF	ADNI (51/52)	10 (10 folds)	82.1	81.9	82.3
Zhang et al., 2011 [20]	PET	ADNI (51/52)	10 (10 folds)	86.5	86.3	86.6
Zhang et al., 2011 [20]	MRI, PET, CSF	ADNI (51/52)	10 (10 folds)	93.2	93.0	93.3
Hinrichs et al., 2011 [31]	MRI + PET	ADNI (48/66)	30 (10 folds)	87.6	78.9	93.8
Hinrichs et al., 2011 [31]	MRI + PET + CSF + APOE + cognitive scores	ADNI (48/66)	30 (10 folds)	92.4	86.7	96.6
Magnin et al., 2009 [24]	MRI	Private (16/22)	5000 (75% training/25% testing)	94.5	91.5	96.6
Klöppel et al., 2008 [22]	MRI	(Group I) private (20/20)	Leave-one-out	95.0	95.0	95.0
Klöppel et al., 2008 [22]	MRI	(Group II) private (14/14)	Leave-one-out	92.9	100	85.7
Klöppel et al., 2008 [22]	MRI	(Group III) private (33/57)	Leave-one-out	81.1	60.6	93.0
Walhovd et al., 2010 [32]	MRI	ADNI (42/38)	N/A	82.5	81.6	83.3
Walhovd et al., 2010 [32]	MRI + CSF	ADNI (42/38)	N/A	88.8	86.8	90.5
Cuingnet et al., 2011* [12]	MRI	ADNI (162/137)	N/A (2 folds)	N/A	81.0	95.0
*Proposed study *	*MRI + MMSE *	*Private (129/60) *	*50 (5 folds) *	*92.3 *	*88.2 *	*94.2 *

*This paper by Cuingnet et al. [12] compares ten methods and the best performance is shown here.

**Table 6:** Univariate analysis of subcortical volumes using different normalization approaches for AD versus CN*.

Volumes	Raw	ICV	Volumes	Raw	ICV
	*P* values			*P* values	
Right hippocampus	<0.00001	<0.00001	Corpus callosum middle anterior	<0.0001	<0.001
Left inferior lateral ventricle	<0.00001	<0.00001	Right accumbens area	<0.0001	<0.01
Left hippocampus	<0.00001	<0.00001	Corpus callosum posterior	<0.0001	<0.01
Left amygdala	<0.00001	<0.00001	Right thalamus proper	<0.0001	<0.01
Right inferior lateral ventricle	<0.00001	<0.00001	Corpus callosum middle posterior	<0.0001	<0.01
Cortex volume	<0.00001	<0.00001	White matter hypointensities	<0.0001	<0.0001
Left hemisphere cortex volume	<0.00001	<0.00001	Left accumbens area	<0.001	<0.001
Right hemisphere-cortex volume	<0.00001	<0.00001	Cerebral spinal-fluid (CSF)	<0.001	<0.00001
Total gray volume	<0.00001	<0.00001	**Right ventral diencephalon**	**<0.001**	**ns**
3rd ventricle	<0.00001	<0.00001	**Left thalamus proper**	**<0.001**	**ns**
Right amygdala	<0.00001	<0.00001	Non-white matter hypointensities	<0.001	<0.01
Right choroid plexus	<0.00001	<0.00001	Subcortical gray volume	<0.01	<0.05
Right lateral ventricle	<0.00001	<0.00001	Optic chiasm	<0.01	<0.01
Left lateral ventricle	<0.00001	<0.00001	5th ventricle	<0.05	<0.05
Left choroid plexus	<0.00001	<0.00001	**Right cerebellum cortex**	**<0.05**	**ns**
Corpus callosum central	*<0.00001 *	*<0.001 *	**Left putamen**	**<0.05**	**ns**
Corpus callosum anterior	<0.00001	<0.00001	Left cerebellum cortex	ns	<0.05

*Two-way Student's *t*-test is used for univariate analysis with a significant level of 0.05 for *P* value.

**Table 7:** Univariate analysis of surface area for left and right hemispheres*.

Surface area normalization	Left hemisphere			Right hemisphere		
	Raw	ICV	Total area	Raw	ICV	Total area
Bankssts	<0.01	<0.05	<0.001	ns	ns	ns
Frontalpole	<0.01	<0.05	<0.05	ns	ns	ns
Paracentral	<0.05	<0.01	<0.01	ns	ns	ns
**Transverse-temporal**	**ns**	**<0.01**	**<0.01**	ns	ns	ns
Lingual	ns	ns	<0.01	ns	ns	ns
**Postcentral**	**ns**	**<0.01**	**<0.01**	ns	ns	ns
**Insula**	**ns**	**<0.05**	**<0.01**	ns	ns	ns
Cuneus	ns	ns	<0.05	ns	ns	<0.05
Temporalpole	ns	ns	ns	<0.01	<0.01	<0.001
**Superior-frontal**	**ns**	**<0.05**	**<0.01**	**ns**	**<0.05**	**<0.01**
**Precentral**	ns	ns	<0.05	**ns**	**<0.05**	**<0.01**
Fusiform	ns	ns	<0.05	ns	ns	<0.01
Inferiortemporal	ns	ns	ns	ns	ns	<0.01
Inferiorparietal	ns	ns	ns	ns	ns	<0.05

*Two-way Student's *t*-test is used for univariate analysis with a significant level of 0.05 for *P* value.

**Table 8:** Univariate analysis of cortical thickness for left and right hemispheres*.

Cortical thickness normalization	Left hemisphere			Right hemisphere		
	Raw	ICV	Mean CT	Raw	ICV	Mean CT
Superiortemporal	<0.00001	<0.00001	<0.00001	<0.00001	<0.00001	<0.00001
Entorhinal	<0.00001	<0.00001	<0.00001	<0.00001	<0.00001	<0.00001
Temporalpole	<0.00001	<0.00001	<0.00001	<0.00001	<0.00001	<0.00001
Inferiortemporal	<0.00001	<0.00001	<0.01	<0.00001	<0.0001	<0.01
Middletemporal	<0.00001	<0.00001	<0.05	<0.00001	<0.0001	<0.05
Parahippocampal	<0.00001	<0.00001	<0.01	<0.00001	<0.00001	<0.05
Fusiform	<0.00001	<0.0001	<0.001	<0.00001	<0.001	<0.01
Supramarginal	<0.00001	<0.0001	ns	<0.00001	<0.001	ns
Lateralorbitofrontal	<0.00001	<0.001	ns	<0.00001	<0.01	ns
Parsorbitalis	<0.00001	<0.001	ns	<0.00001	<0.001	ns
Bankssts	<0.00001	<0.0001	ns	<0.00001	<0.001	ns
Superiorfrontal	<0.00001	<0.001	<0.05	<0.00001	<0.001	ns
Parsopercularis	<0.00001	<0.001	ns	<0.00001	<0.01	ns
Insula	<0.00001	<0.001	<0.01	<0.00001	<0.001	<0.01
Rostralanteriorcingulate	<0.00001	<0.01	<0.05	<0.00001	<0.001	<0.001
Isthmuscingulate	<0.00001	<0.01	ns	<0.00001	<0.001	ns
Inferiorparietal	<0.00001	<0.001	<0.05	<0.00001	<0.001	ns
Transversetemporal	<0.00001	<0.001	ns	<0.001	<0.05	ns
Caudalanteriorcingulate	<0.00001	<0.01	ns	<0.00001	<0.01	ns
Parstriangularis	<0.00001	<0.01	<0.05	<0.00001	<0.01	<0.01
Rostralmiddlefrontal	<0.00001	<0.05	<0.0001	<0.00001	<0.05	<0.01
Caudalmiddlefrontal	<0.00001	<0.01	ns	<0.00001	<0.01	ns
Posteriorcingulate	<0.00001	<0.01	ns	<0.00001	<0.01	ns
Precuneus	<0.00001	<0.01	ns	<0.00001	<0.01	ns
**Medialorbitofrontal**	<0.00001	<0.05	ns	**<0.001**	**ns**	**ns**
Precentral	<0.00001	<0.05	<0.05	<0.0001	<0.05	ns
**Frontalpole**	<0.0001	<0.05	ns	**<0.01**	**ns**	**ns**
Postcentral	<0.01	ns	<0.00001	<0.01	ns	<0.00001
Superiorparietal	<0.01	ns	<0.00001	<0.01	ns	<0.0001
Lateraloccipital	<0.01	ns	<0.00001	<0.05	ns	<0.00001
Lingual	<0.05	ns	<0.00001	<0.01	ns	<0.00001
Paracentral	<0.05	ns	<0.01	<0.01	ns	<0.01
Pericalcarine	ns	ns	<0.00001	ns	ns	<0.00001
Cuneus	ns	ns	<0.00001	ns	ns	<0.00001

*Two-way Student's *t*-test is used for univariate analysis with a significant level of 0.05 for *P* value.
